# Definitive Treatment of Brain Metastases From a Neuroendocrine Tumor With Peptide Receptor Radionuclide Therapy With 177Lutetium DOTATATE: A Case Report

**DOI:** 10.7759/cureus.45327

**Published:** 2023-09-15

**Authors:** Vivian Zhang, Kekoa Taparra, George Fisher, Carina Aparici, Scott G Soltys

**Affiliations:** 1 Department of Radiation Oncology, Stanford University, Stanford, USA; 2 Department of Medicine and Medical Oncology, Stanford University, Stanford, USA; 3 Department of Nuclear Medicine and Molecular Imaging, Stanford University, Stanford, USA

**Keywords:** response, prrt, net, neuroendocrine tumor, brain metastases

## Abstract

Gastroenteropancreatic neuroendocrine tumors (GEP-NETs) are rare malignancies that arise from secretory endocrine cells of the gastroenteropancreatic system. Clinical outcomes have improved for patients with GEP-NETs due to the development and recent FDA approval of ^177^Lutetium DOTATATE. However, the response of brain metastases from GEP-NETs from ^177^Lutetium DOTATATE is unreported.

We present the case of an 81-year-old man with low-grade small bowel GEP-NET with liver and brain metastases treated with a total of six cycles of ^177^Lutetium DOTATATE. With over three years of follow-up from his initial treatment, his brain metastases have had complete or partial responses, with no need for brain radiotherapy or radiosurgery.

## Introduction

Neuroendocrine tumors (NETs) are a rare and heterogeneous group of malignancies that arise from secretory endocrine cells. The age-adjusted incidence of NETs per 100,000 has risen from 1.1 in 1973 to 7.0 in 2012 [1]. Neuroendocrine tumors most commonly occur in the gastroenteropancreatic (GEP) system (68%), with small bowel NETs being the most common GEP-NETs (42%) [2]. Brain metastases are rare in patients with NET and are associated with a poor prognosis of 10 months of average overall survival [3]. The estimated incidence of brain metastases from NETs is 1.5%-5% [4]. The standard treatment options for any patient with brain metastasis, including from NETs, include stereotactic radiosurgery (SRS), whole brain radiotherapy (WBRT), surgery, and/or systemic therapy if it has activity within the central nervous system (CNS).

^177^Lutetium DOTATATE is a peptide receptor radionuclide therapy (PRRT) that targets and enters cells expressing somatostatin type-2 receptors (SST2R) to deliver radiation. Since its approval by the FDA in 2018 for the treatment of GEP-NETs, limited data exist reporting the CNS activity and response of brain metastases from PRRT. In this Stanford University Institutional Review Board-approved retrospective review, we present a patient with GEP-NET with brain metastases who was successfully and durably treated with ^177^Lutetium DOTATATE.

## Case presentation

An 81-year-old man with a history of grade 1 ileal NET metastatic to the liver and brain was referred to the radiation oncology clinic for consideration of treatment of new brain metastases. He initially presented in 2001 with a guaiac-positive stool, which led to a colonoscopy identifying an obstructive lesion. He underwent laparoscopic surgery and was converted to open laparotomy with terminal ileum resection and right hemicolectomy. He was initially staged as a T3 N2 M1a well-differentiated G1 neuroendocrine tumor with six of 12 lymph nodes involved. A biopsy of liver lesions later revealed a metastatic carcinoid tumor. Resection was deferred due to the high tumor burden and received four months of everolimus and octreotide on a phase II trial in 2005 [5], discontinued after four months due to cytopenic toxicity.

He remained clinically and radiographically stable on surveillance from 2005 to 2009. In 2009, imaging showed stable hepatic disease but new right upper lobe pulmonary nodules. Given that these were asymptomatic, he continued surveillance without active therapy. In 2016, he developed carcinoid symptoms and re-started monthly octreotide injections. In 2018, due to interval growth of his hepatic metastases, he had two courses of Yitrium-90 hepatic radioembolization, with subsequent shrinkage of the lesions.

**Figure 1:**
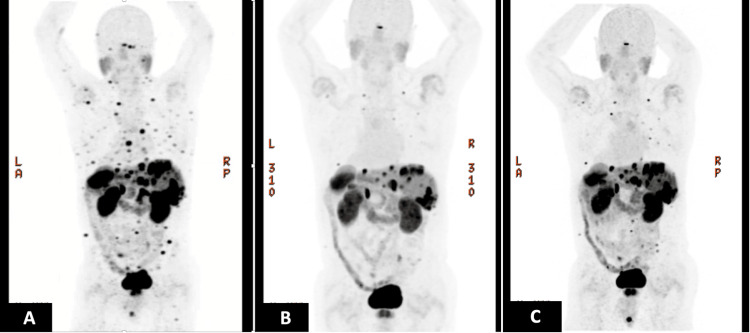
The patient's DOTATATE PET/CT prior to (A) and after four cycles (B) of 200 mCi 177Lutetium DOTATATE peptide receptor radionuclide therapy (PRRT) displayed complete and partial responses in all extracranial metastases. Twenty-five months later (C), he had progression in extracranial sites, treated with additional cycles of PRRT; his brain remained controlled at the time of this extracranial progression. PRRT: peptide receptor radionuclide therapy

In 2019, for symptoms of right vision loss and blurriness, a brain MRI revealed orbit metastases as well as nine small brain metastases. A DOTATATE PET/CT showed widespread metastatic disease of the liver, bone, lymph nodes, and soft tissue, as well as the bilateral orbits and brain (Figure 1A).

**Figure 2:**
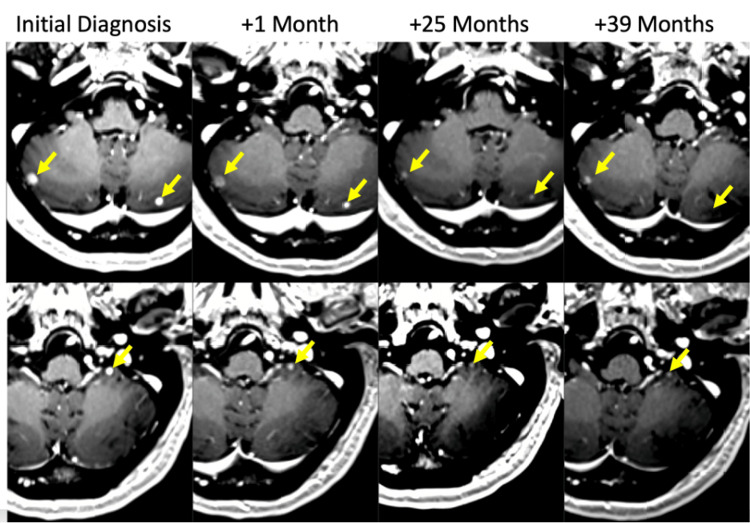
Rapid and durable response following PRRT, with deferral of brain radiotherapy or radiosurgery, of multiple cerebellar brain metastases from GEP-NET shown are representative images of three of nine total cerebellar metastases at the time of initial diagnosis and at one month following the start of PRRT; an early response is seen. These responses were durable at 25 months post-diagnosis; at this time, two additional cycles of PRRT were given due to systemic progression. The most recent MRI at 39 months displays continued stability of the treated brain metastases, with no new brain metastases. PRRT: peptide receptor radionuclide therapy; GEP-NET: gastroenteropancreatic neuroendocrine tumors

A CT-guided liver biopsy again confirmed well-differentiated, grade 1 (KI-67 1% and 1 mitosis/10 high power fields) NET. A repeat brain MRI two months later, after establishing care with Stanford Health Care, showed interval growth of the brain metastases (e.g., right cerebellar from 4.7 x 4.7 mm to 6.5 x 5.1 mm and left cerebellar from 2.8 mm to 4.0 mm) (Figure 2).

The patient was referred to radiation oncology to discuss radiosurgery or radiotherapy for his brain metastases. Given his extra-cranial progression, it was planned to start ^177^Lutetium DOTATATE (Lutathera) treatment given the results from the Neuroendocrine Tumors Therapy (NETTER-1) trial [6], which showed a significantly longer progression-free survival and higher response rate than high-dose octreotide in patients with advanced midgut NET. At this time, alpha emitters such as Ac-DOTATOC were not yet FDA-approved. Although SRS was the standard option for his brain metastases, given his orbit metastases, whole-brain radiotherapy may have been required to treat both the brain and the orbits. Given that he was asymptomatic, with no associated edema, and his vision was stable over many months from the orbit metastases, the patient was presented with the option to see if the planned PRRT could have an intracranial response in the brain (on the brain side of the blood-brain barrier) and in the orbits (on the systemic side of the blood-brain barrier). The patient preferred to defer SRS to the brain and was followed up with repeat brain MRIs every month during his PRRT.

He received four cycles of 200 mCi ^177^Lutetium DOTATATE treatment from February 2020 to August 2020. Repeat DOTATATE PET/CT showed an overall decrease in his widespread systemic metastases (Figure 1B). An MRI one month following the start of PRRT showed stability or early response in his previously growing brain and orbital metastases (Figure 2). Near the end of the four cycles, his brain MRI showed continued shrinkage in the largest lesions (e.g., right cerebellar from 6.5 x 5.1 mm to 5.4 x 4.8 mm and left cerebellar from 4.0 mm to 3.6 mm) and stability in the smaller metastases.

Although his brain remained stable 25 months later (Figure 2), his March 2022 DOTATATE PET showed progression in liver, bone, and lung lesions (Figure 1C). Therefore, he received two additional cycles of 200 mCi ^177^Lutetium DOTATATE treatment in June 2022, with a partial response to his systemic disease. Considering his age, peritoneal disease, and brain metastases, he was provided a two-week taper of dexamethasone (4 mg) after each cycle. Fatigue after each treatment was more obvious and lasted longer than his previous PRRT treatments. He experienced no headaches, vomiting, or diarrhea, and his energy returned to normal two weeks post-treatment. These six cycles of treatment were well tolerated, with the highest level of toxicity (per the Common Terminology Criteria for Adverse Events (CTCAE v4.0)) being grade 3 hypertension (history of labile hypertension, adjusted blood pressure medication with better control, no delays in PRRT treatment), grade 2 fatigue, and grade 1 nausea.

Since the initial response in his brain metastases to four cycles of PRRT, he has had stability in all brain MRIs from 2021 through 2023, with no progression of previous sites and no new metastases. Of note, at the time of his additional two cycles of PRRT due to systemic progression, he did not have intracranial progression. Similarly, no further shrinkage of any brain metastasis was seen with the additional cycles of PRRT. With 39 months of follow-up surveillance brain MRIs from 2021 to 2023, he has continued control and persistently resolved metastatic brain disease without recurrence or progression (Figure 2). His most recent 2023 MRI of the abdomen and pelvis showed stable disease with no new lesions, and his right blurred vision, attributed to progressive glaucoma, remained stable too with prescription eye drops. In our patient, it appears that PRRT achieved durable local control of brain metastases as the definitive treatment, with deferral of radiosurgery or radiotherapy.

## Discussion

We present a patient with metastatic GEP-NET to the brain that responded to ^177^Lutetium DOTATATE treatment, resulting in a long-term, durable response to his brain metastases with no need for brain radiotherapy or radiosurgery. Brain metastases from primary gastrointestinal (GI) neuroendocrine tumors have a poor prognosis, with a reported average overall survival of only ten months [3]. Our patient is alive and well, with over three years since his initial diagnosis of brain metastases. Our report is notable as it highlights the potential for a rapid and durable intracranial response of brain metastases from PRRT with ^177^Lutetium DOTATATE.

Based on the published literature, there is no consensus on the standard of treatment for intracranial metastases from NETs. Treatment options for any patient with brain metastases of any histology include whole-brain radiation therapy, stereotactic radiosurgery, surgical resection, and/or CNS-penetrant systemic therapy. In a retrospective 24-patient series from 2004, the median overall survival of patients with NET brain metastases appeared greater in those with aggressive treatment with combined surgery followed by WBRT (3.2 years) compared to surgery alone (4.8 months) or WBRT alone (six months) [3]. In contrast, a retrospective 51-patient series found no significant difference in overall survival between patients treated with WBRT, surgery plus WBRT, or observation using an overall long-rank test (p=0.72) [7].

^177^Lutetium DOTATATE is a PRRT somatostatin analog that selectively delivers radiation to cells expressing SST2R. Previous and ongoing clinical trials support the clinical benefit of ^177^Lutetium DOTATATE for treating NETs. A phase 1 clinical trial completed in 2017 showed that ^177^Lutetium DOTATATE plus nivolumab in patients with small cell lung cancer or advanced lung NET was well tolerated and showed signs of antitumor activity [8]. In the NETTER-1 phase III clinical trial, 229 patients with well-differentiated, metastatic midgut NETs were randomly assigned to treatment with ^177^Lutetium DOTATATE plus octreotide long-acting repeatable (LAR) compared to octreotide LAR alone (control group) [6]. The estimated progression-free survival (PFS) at 20 months was 65% (95% confidence interval [CI], 50.0 to 76.8) for those treated with ^177^Lutetium DOTATATE, compared to 11% (95% CI, 3.5 to 23.0) for the control group (p<0.001). The response rate was 18% for ^177^Lutetium DOTATATE versus 3% in the control group (p<0.001). With long-term follow-up, the median overall survival was 48.0 months (95% CI 37.4-55.2) for ^177^Lutetium DOTATATE versus 36.3 months (95% CI 25.9-51.7) in the control group (p=0.30) [9].

In 2018, the FDA approved ^177^Lutetium DOTATATE for treating adults with GEP-NETs expressing SST2R. Gastroenteropancreatic neuroendocrine tumors express one of the highest densities and frequencies (80%-100% of cases) of the SST2R subtype among all somatostatin receptor-expressing tumors [10]. In addition, somatostatin receptors are generally expressed homogenously in GEP-NETs. These characteristics support the clinical benefit of somatostatin analogs like ^177^Lutetium DOTATATE for treating GEP-NETs. More data are needed to report intracranial responses to treatment; we await the reporting of an ongoing phase I/II trial of ^177^Lutetium DOTATATE in CNS tumors expressing SST2R, including meningioma, medulloblastoma, malignant glioma, and brain metastases of neuroendocrine tumors [11].

The diagnosis of brain metastasis significantly impacts the patient's prognosis. The five-year survival rate for metastatic NET excluding the brain is 40%, while the average overall survival for brain metastasis from NET is less than a year [3]. Like with other histologies metastatic to the brain, neurologic death is still less common than organ dysfunction due to systemic disease progression [12,13]. There may be differences between intracranial and systemic control with ^177^Lutetium DOTATATE. In general, the systemic response of chemotherapy for GEP-NETs usually lasts less than one year [14]. Our patient developed systemic progression in the liver, bone, and lung one year and seven months post-PRRT while the brain remained stable.

## Conclusions

Although brain metastases from NETs are rare, their diagnosis portends a poor prognosis. While systemic responses from ^177^Lutetium DOTATATE are well documented, we are unaware of data reporting intracranial responses of brain metastases to PRRT. To our knowledge, this may be the first case report of a gastroenteropancreatic neuroendocrine tumor metastatic to the brain that achieved complete or partial responses following ^177^Lutetium DOTATATE. For over three years following his first course of PRRT treatment, his brain metastases have been durably controlled, with no new or progressive brain metastases, with the omission of standard brain irradiation therapy. To improve the care and options for our patients, we encourage the reporting of additional patients treated with PRRT for NET brain metastases.
